# Effects of *Ficus umbellata* (Moraceae) Aqueous Extract and 7-Methoxycoumarin on Scopolamine-Induced Spatial Memory Impairment in Ovariectomized Wistar Rats

**DOI:** 10.1155/2018/5751864

**Published:** 2018-09-30

**Authors:** Stéphane Zingue, Harquin Simplice Foyet, Sefirin Djiogue, Yannick Ezo'o Ezo'o, Hervé Hervé Ngatanko Abaïssou, Pafroumi Fachagbo, Dieudonné Njamen

**Affiliations:** ^1^Department of Life and Earth Sciences, Higher Teachers' Training College, University of Maroua, P.O. Box 55, Maroua, Cameroon; ^2^Department of Animal Biology and Physiology, Faculty of Science, University of Yaoundé 1, P.O. Box 812, Yaoundé, Cameroon; ^3^Department of Biological Sciences, Faculty of Science, University of Maroua, P.O. Box: 814, Maroua, Cameroon

## Abstract

The present work was undertaken to evaluate the ability of *F. umbellata* aqueous extract and its major component 7-methoxycoumarin (MC) to improve scopolamine-induced spatial memory impairment in ovariectomized Wistar rats. For this to be done, 10 sham-operated and 30 postmenopausal-like rats were randomly distributed in eight groups (*n* = 5) and treated with distilled water (2 mL/250 g), estradiol valerate (1 mg/kg BW), piracetam (1.5 mg/kg BW), *F. umbellata* aqueous extract (50 and 200 mg/kg BW), or MC (1 mg/kg BW) for 21 consecutive days. Before and after the memory impairment with scopolamine (2 mg/kg BW), animals underwent behavioral evaluations on Y- and radial mazes. As results, age and ovariectomy did not induce significant changes in the reference memory errors. While age decreased working memory errors, ovariectomy increased it. The MC as well as *F. umbellata* extract significantly increased (*p* < 0.01) the percentage of spontaneous alternation and decreased (*p* < 0.001) working and spatial reference memory errors and anxiety parameters (rearing and grooming) in ovariectomized rats. MC significantly reduced (*p* < 0.05) the MDA level, but resulted in an increase in GSH level in brain homogenates. These results suggest that MC is endowed with neuroprotective effects and could account for the neuroprotective effects of *F. umbellata* in rats.

## 1. Introduction

Estrogens are steroidal hormones mainly produced by ovaries, which greatly fluctuate during women's lifespan and decline with menopause [1]. Numerous studies indicate that estrogens have neuroprotective effects either by direct activation of estrogen receptors (ERs), which are located in the brain, or by activation of receptors of other neuroprotective factors [1]. Studies in female rodents have shown that a decrease in plasmatic estrogen levels following ovariectomy enhances brain damage under neurodegenerative conditions [2, 3] and increases amyloid-*β* oligomers in a mouse model of Alzheimer's disease (AD) [4]. AD is the most frequent form of dementia and is an age-related neurodegenerative disease characterized by a loss or decline in memory and severe cognitive as well as behavioral impairments [5]. According to the World Alzheimer Report [6], 46.8 million people worldwide have dementia, out of which 60 to 70% are AD patients. To date, the pathophysiology of AD remains unclear, but it is considered that the *β*-amyloid and tau protein aggregation, reduced acetylcholine (ACh), and glutamatergic deficit are principal agents of the pathogenesis of AD [7]. However, age, genetic predisposition, stress, emotions, and the sudden drop in estrogen levels after menopause are nowadays recognized as risk factors that may contribute to AD symptoms [8, 9].

Hormone replacement therapy (HRT) has been used for many years to treat symptoms of menopause, including hot flushes and depression. Prospective studies have shown that it enhances cognitive skills in postmenopausal women [10]. Nevertheless, a long-term use of HRT is associated with adverse effects such as increased risks of endometrial and breast cancers, stroke, and pulmonary thromboembolism [11–13]. Hence, many women resort to natural health remedies as alternative therapies to treat menopausal symptoms [14]. Plant preparations and plant-derived compounds (phytoestrogens) are being considered as natural alternative to hormone replacement therapy.

*Ficus umbellata* (Moraceae) is a tropical tree commonly called “Mewed” in “Guiziga.” Its back is used in indigenous medicine for the treatment of menopause-related physiological disorders [15]. Just like *F. sur*, *F. platyphylla*, *F. ovata*, *F. ingens*, and *F. glumosa*, it is used to treat some nervous system disorders such as dementia, neuralgia, and epilepsy [15, 16]. Previous studies revealed the transactivation of ER*α* in a reporter gene assay induces significant estrogen-like effects on estrogen target organs (uterus, vagina, and mammary gland) in rats when treated with *F. umbellata* aqueous and methanol extracts. These extracts also brought about a significant decrease in the frequency of occurrence of hot flushes in experimental rats. The major component of these extracts after isolation and characterization was found to be 7-methoxycoumarin (MC) [17]. Additionally, MC exhibited estrogen-like and tissue-selective effects in vitro on cell-based assay and in vivo on Wistar rats [18]. In the present study, the neuroprotective potential of MC as well as *F. umbellata* aqueous extract was evaluated using scopolamine-induced spatial memory impairment in ovariectomized Wistar rats. The investigation focused on number of entries, the percentage of spontaneous alternation, and working and spatial reference memory errors. Since scopolamine-induced memory deficits are accompanied with oxidative damage, oxidative stress biomarkers (MDA and GSH levels) were also assessed.

## 2. Materials and Methods

### 2.1. Chemicals

Estradiol valerate (Progynova® 2 mg) was purchased from Delpharm (Lille, France). UPLC-grade solvents methanol, acetonitrile (ACN), methyl tert-butyl ether (MTBE), water, scopolamine, piracetam, and formic acid (FA) were purchased from Sigma-Aldrich (Saint-Quentin-Fallavier, France). Penicillin (xtapen®) was provided by CSPC Zhongnuo Pharmaceutical (Shijiazhuang City, China). Diclofenac (Dicloecnu®) was provided by ECNU Pharmaceutical (Yanzhou City, China).

### 2.2. Plant Material and Preparation of *F. umbellata* Extracts

Stem bark of *F. umbellata* was harvested in Yaounde (centre region, Cameroon) in September 2013. This botanical sample was authenticated by Mister Victor Nana, botanist at the National Herbarium of Cameroon (HNC) by comparison to the specimens deposited under the voucher number 99/HNC. After shade drying and grinding, 2000 g of powder was macerated in water at room temperature (5 L of solvent × 3, 48 h per extraction). The combined solutions were filtered with Whatman paper number 4 and evaporated using an oven with ventilation (40°C, during 48 h) to yield 229.8 g of aqueous crude extract. Alternatively, 2700 g of the powder was macerated in 95% methanol at room temperature (5 L of solvent × 3, 48 h per extraction). The combined solutions were evaporated under reduced pressure (337 mbar at 40°C) using a rotary evaporator to yield 162 g of a methanol crude extract (MeOH).

### 2.3. Isolation of 7-Methoxycoumarin (MC)

**Figure 1:**
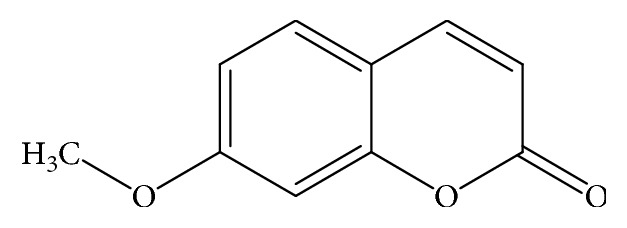
7-Methoxycoumarin (molecular weight = 176.1 M; molecular formula = C_10_H_8_O_3_) [17].

UPLC chromatograms of *F. umbellata* aqueous as methanol extracts showed that MC is their major compound (Supplementary Data 1). After a bioguided fractionation of *F. umbellata* methanol extract, MC was isolated [17].  Figure 1 shows a summary of the physicochemical properties of MC.

### 2.4. Animals

Healthy young female Wistar rats aged four weeks, weighing (~ 150 g), were used in this study. These rats were purchased from the breeding facility of the Animal Physiology Laboratory of the University of Yaounde I (Cameroon). Animals were housed in clean polyacrylic cages in a light-controlled room (approx. 12 h light/dark), and temperature was maintained around 25°C. A soy-free rat chow diet was provided to the animals with water ad libitum. The composition of animal diet was as follows: corn (36.7%), bone flour (14.5%), wheat (36.6%), fish flour (4.8%), crushed palm kernel (7.3%), sodium chloride (0.3%), and vitamin complex (Olivitazol® 0.01%). Housing of animals and all experiments were conducted in accordance with the principles and procedures of the European Union legislation on the use of animals for scientific purposes (Directive 2010/63/EU) adopted by the Cameroon Institutional National Ethic Committee, Ministry of Scientific Research and Technology Innovation (reg. number FWA-IRD 0001954).

### 2.5. Study Design

At the beginning of the experiment, 5 rats aged 2.5 months underwent behavioral evaluations on Y- and radial mazes in order to record basic data (BD). Afterwards, ketamine (50 mg/kg BW; ip) and diazepam (10 mg/kg BW; ip) anesthesia were used. 30 rats were bilaterally ovariectomized using the dorsal approach, and 10 others were sham-operated. Animals were allowed for (84) days for the setup of menopausal-like conditions [19–21], followed by the random distribution of animals (now aged 5.5 months) into eight groups (*n* = 5). Groups I (SHAM 1) and II (SHAM 2) were sham-operated rats and received distilled water. Groups III to VIII were ovariectomized rats treated as follows: Group III received distilled water and served as negative control (OVX). Group IV served as positive control 1 and received estradiol valerate (1 mg/kg BW). Group V served as positive control 2 and received piracetam, a nootropic drug that exhibits cognition-enhancing properties [22] at a dose of 1.5 mg/kg BW. Groups VI and VII received the *F. umbellata* aqueous extract at the doses of 50 and 200 mg/kg, respectively. Group VIII received the MC at the dose of 1 mg/kg. Distilled water was used as a vehicle for the dissolution of substances which were administered orally using a feeding tube (2 mL/250 g) for three weeks (a single dose per day). Piracetam and MC were given intraperitoneally (0.3 mL/250 g). However, parallel injections/gavages of vehicle were given so that all rats had the same administration experiences. Behavioral testing was achieved between the 12th and 21st days of treatment. On the last day of treatment, scopolamine (2 mg/kg) was administered as a single dose 30 min after drug administration through intraperitoneal (ip) injection route to all the groups except group I (SHAM 1). Exceptionally this day, behavioral testing was done before and after scopolamine injection. A right hemisphere of each animal's brain was homogenized to give 20% (*w*/*v*) homogenate in an ice-cold sodium buffer (0.1 M, pH 7.3). The homogenate was centrifuged at 3000 ×g for 15 min at 4°C. The supernatant was stored at −20°C for the determination of some oxidative stress biomarkers.

### 2.6. Behavioral Testing

#### 2.6.1. Y-Maze Test

Short-term memory was assessed by spontaneous alternation behavior in the Y-maze task as previously reported by Foyet et al. [23]. Briefly, the maze consisted of three arms (35 cm long, 25 cm high, and 10 cm wide) and an equilateral triangular central area. Each rat was placed in a randomized order at the end of one arm and allowed to move freely through the maze for 8 min. An arm entry was counted when the hind paws of the rat were completely within the arm. Spontaneous alternation behavior was defined as three consecutive entries in three different arms (i.e., A, B, C or B, C, A, etc.). The percentage alternation score (PAS) was calculated by the following formula: PAS = [total alternation number/(total number of entries − 2)] × 100. Furthermore, the total number of arm entries was used as a measure of general activity of the animals. The maze was wiped clean with 70% ethanol between each animal to minimize odor cues [23, 24].

Anxiety negatively influences learning activity and new information storage; some anxiety parameters such as frequency of rearing and grooming [25] were therefore recorded during the Y-maze test.

#### 2.6.2. Radial Arm Maze

In this study, the radial arm maize was used to assess working memory and reference memory as previously reported by Hritcu et al. [26]. This apparatus was a wooden platform raised 50 cm above the floor, consisting of 8 arms (48 cm long × 12 cm wide). All the arms numbered 1–8 extended radially from a central area (32 cm in diameter). A food cup containing a single 50 mg food pellet was placed at the end of each arm. Visual cues were placed around the maze and maintained in their positions throughout the testing period. In order to maintain at 85% of their free feeding weight, the animals were kept on restricted diet over a week prior to the performance of the maze task with free access to water ad libitum. Before the trial training for recording sequences of alternation in the radial maze arms, three rats were placed in the central platform of the radial maze and allowed to explore freely and consume the food distributed on the central platform and arms. The food was progressively restricted to the arms and the food cups. During the trial training, a rat is placed on the central platform and allowed to explore until all the 5 baits had been consumed or until 5 minutes has elapsed. During the eight-day tests, each animal is placed individually on the central platform of the radial maze facing an arm for the assessment of reference memory and working memory. Five arms (numbers 1, 2, 4, 5, and 7) out of 8 were baited throughout the training days. The number of reference memory errors (entering an arm that was not baited) and working memory errors (entering an arm containing food, but previously entered) was measured. An arm entry was counted when all the four limbs of the animal were within the arm. 70% ethanol was used to clean the maze after the passage of each rat in order to reduce residual odors [26, 27].

### 2.7. Histological Analysis

The histomorphology of the brain was performed from 5 *μ*m sections of paraffin-embedded tissues following hematoxylin-eosin staining. Brains were photographed at 40x magnification using the complete Zeiss equipment consisting of a microscope Axioskop 40 connected to a computer where the image was transferred, and analyzed with the MRGrab1.0 and AxioVision 3.1 software, all provided by Zeiss (Hallbergmoos, Germany).

### 2.8. Biochemical Analysis

#### 2.8.1. Total Content of Reduced Glutathione GSH

To measure the reduced glutathione (GSH) level, the brain homogenate in sodium buffer (0.1 M, pH 7.3) was taken. The procedure was followed as previously described by Shamnas et al. [28]. Briefly, the homogenate was mixed with an equal volume of trichloroacetic acid (TBA, 20%) containing 1 mM EDTA to precipitate the proteins. The mixture was allowed to stand for 5 minutes prior to centrifugation. The supernatant (200 *μ*L) was then transferred to a new set of test tubes to which was each added 1.8 mL of Ellman's reagent (5,5′-dithiobis*-*2-nitrobenzoic acid) (0.1 mM) prepared in 0.3 M phosphate buffer with 1% sodium citrate solution. Then, the content in all the test tubes was made up to the volume of 2 mL with distilled water. After the completion of the total reaction, the solutions were measured at 412 nm against a blank. Absorbance values were then compared with a standard curve generated from GSH.

#### 2.8.2. Determination of MDA Level

Malondialdehyde (MDA), which is a measure of lipid peroxidation, was spectrophotometrically measured using the thiobarbituric acid assay as reported by Padurariu et al. [29]. Briefly, 200 *μ*L of brain homogenate was added and mixed with 1 mL of 50% trichloroacetic acid in 0.1 M HCl and 1 mL of 26 mM thiobarbituric acid. After vortex mixing, samples were maintained at 95°C for 20 minutes. Furthermore, samples were centrifuged at 800 ×g for 10 minutes and supernatants were read at 532 nm. A calibration curve was constructed using MDA as standard, and the results were expressed as mM/mg protein.

### 2.9. Statistical Analysis

Results were presented as means ± standard error of the mean (SEM). All formula and functions were calculated with Microsoft Excel software. Data analysis was performed with GraphPad Prism 5.0 software. The normal distribution of data was assessed by Shapiro-Wilk test, and the distribution was found normal (*p* = 0.5). Further, the one-way ANOVA test was followed by the Newman-Keuls post hoc test; a stepwise multiple comparison procedure was used to identify sample means that are significantly different from each other. Differences were considered significant at a probability level of 5% (*p* < 0.05).

## 3. Results

### 3.1. Effect of Age and Ovariectomy on Short-Term and Long-Term Memories

**Figure 2:**
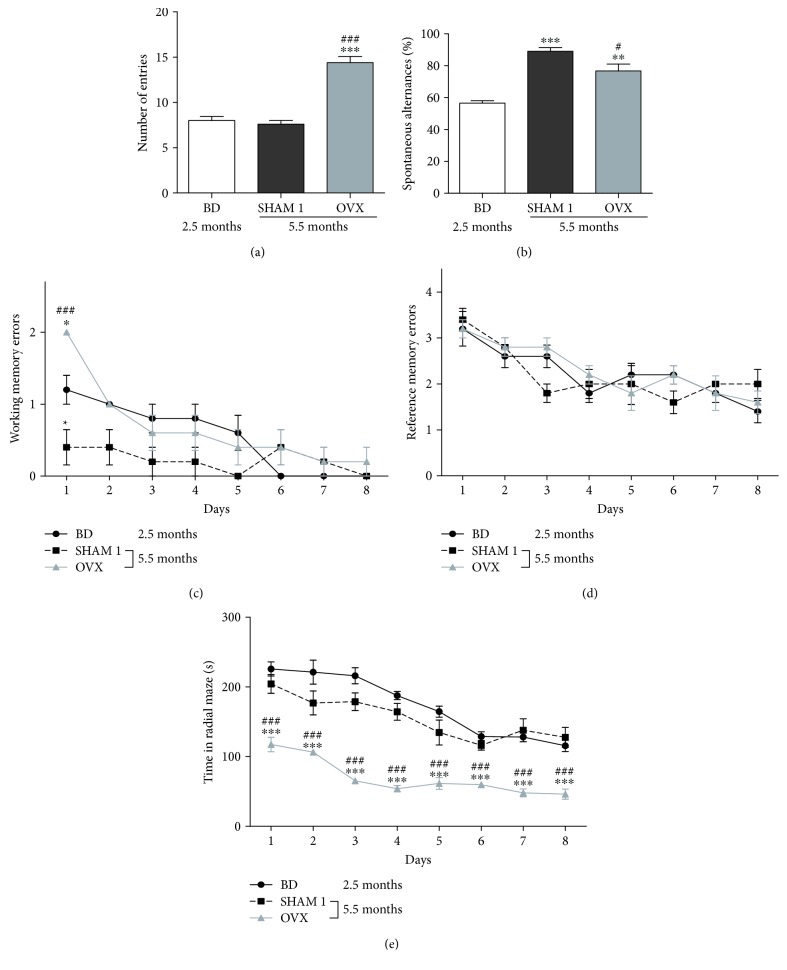
Effects of age and ovariectomy on number of entries (a), spontaneous alternances (b), working memory (c) and spatial reference memory (d) errors, and the time past in radial maze (e). OVX = ovariectomized rat aged 5.5 months; BD = nonovariectomized rats aged 2.5 months which served as basic data; SHAM 1 = nonovariectomized rats aged 5.5 months. ^∗^ *p* < 0.05, ^∗∗^ *p* < 0.01, and ^∗∗∗^ *p* < 0.001 as compared to control BD. ^#^ *p* < 0.05 and ^###^ *p* < 0.001 as compared to the NOR group.

As observed in Figure 2, age has no effect on number of entries (Figure 2(a)) in the Y-maze. Indeed, no significant change was noted in normal rats aged 5.5 months (SHAM 1) as compared to the basic data (BD) recorded in youngest animal (2.5 months).  Figure 2(b) shows that the percentage of spontaneous alternation behavior significantly (*p* < 0.001) increased with age. Furthermore, ovariectomy significantly (*p* < 0.001) increased the number of entries (Figure 2(a)) but significantly (*p* < 0.05) reduced the percentage of spontaneous alternation as compared to the age-matched normal animal (SHAM 1).

Age and ovariectomy did not induce significant changes in the reference memory errors (Figure 2(d)), while normal rats aged 5.5 months made less working memory errors than the youngest ones did (2.5 months), although significant (*p* < 0.01) only on day 1 of the experiment (Figure 2(c)). The ovariectomized rats made more working memory errors as compared to the age-matched normal rats (Figure 2(c)). Interestingly, the ovariectomized rats took a significantly (*p* < 0.001) lower time to collect baits as compared to the normal group (Figure 2(e)). It is worth noting that working memory error and reference memory error as well as the time taken to collect all baits decreased continuously throughout the experiment in all groups.

### 3.2. Effect of Age and Ovariectomy on Anxiety Parameters

**Figure 3:**
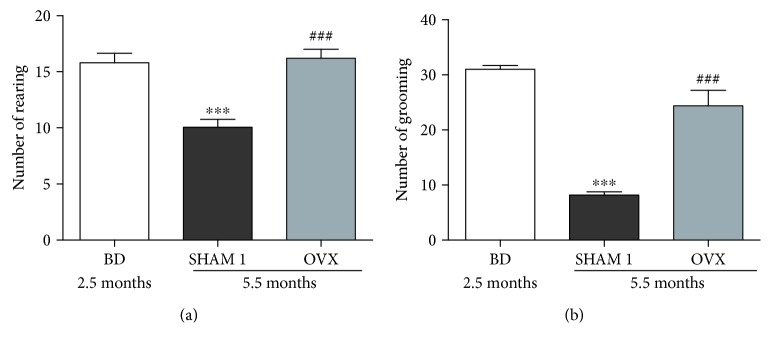
Effects of age and ovariectomy on number of rearing (a) and the number of grooming (b) of rats in the Y-maze. OVX = ovariectomized rat aged 5.5 months; BD = nonovariectomized rats aged 2.5 months which served as basic data; SHAM 1 = nonovariectomized rats aged 5.5 months. ^∗∗∗^ *p* < 0.001 as compared to control BD. ^###^ *p* < 0.001 as compared to the NOR group.

The number of rearing and grooming significantly (*p* < 0.001) decreased in normal rats aged 5.5 months as compared to youngest rats (2.5 months) (Figure 3). Ovariectomy significantly (*p* < 0.001) increased these parameters as compared to aged-matched normal rats (SHAM 1).

### 3.3. Effects of 7-Methoxycoumarin and *F. umbellata* Extract on Short-Term Memory

**Figure 4:**
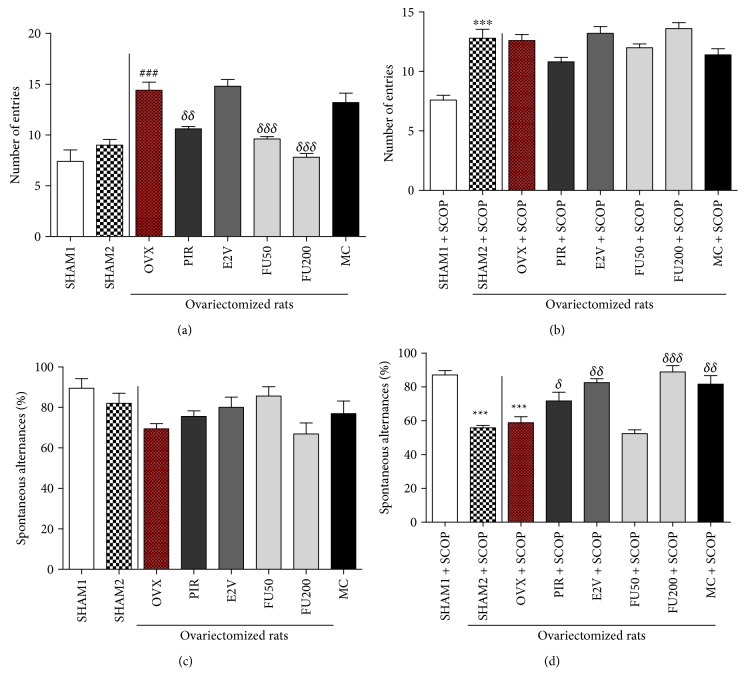
Graphic representation of the number of entries (a and b) and percentage of spontaneous alternances (c and d) before and after scopolamine exposition in rats treated with different substances into the Y-maze. SHAM 1 = sham-operated rats treated with vehicle as normal control 1; SHAM 2 = sham-operated rats treated with vehicle as normal control 2; OVX = ovariectomized rats treated with vehicle as negative control; PIR = ovariectomized rats treated with piracetam at the dose of 1.5 mg/kg BW as positive control 1; E2V = ovariectomized rats treated with estradiol valerate at the dose of 1 mg/kg BW as positive control 2; FU 50 and 200 = ovariectomized rats treated with *F. umbellata* aqueous extract at the doses of 50 and 200 mg/kg BW, respectively; MC = ovariectomized rats treated with 7-methoxycoumarin at the dose of 1 mg/kg BW. SCOP = animal that received a single dose of scopolamine (2 mg/kg BW). ^∗∗∗^ *p* < 0.001 as compared to the SHAM 1 group; ^###^ *p* < 0.001 as compared to the SHAM 2 group; ^*δ*^ *p* < 0.01, ^*δδ*^ *p* < 0.01, and ^*δδδ*^ *p* < 0.001 as compared to the OVX group.

Before memory impairment with scopolamine, ovariectomized rats (OVX) had a significantly higher (*p* < 0.001) number of entries into the arms of the Y-maze than normal rats (SHAM 2). *F. umbellata* aqueous extract treatment at both doses (50 and 200 mg/kg) significantly (*p* < 0.001) reduced this parameter just like piracetam (1.5 mg/kg) as compared to the OVX group (Figure 4(a)). Thirty minutes after injection of scopolamine, the number of entries significantly (*p* < 0.001) increased in normal rats treated with scopolamine (SHAM 2 + SCOP) as compared to the age-matched normal group, which did not receive scopolamine (SHAM 1). No significant change was observed between all animals to which scopolamine was administered (Figure 4(b)).

Figure 4(c) shows a nonsignificant decrease in the percentage of spontaneous alternations behavior with ovariectomy before the administration of scopolamine. There was a nonsignificant increase in this parameter in all OVX rats treated with different substances (except with FU 200). After memory impairment (Figure 4(d)), a significant decrease in the spontaneous alternations was observed in normal rats (SHAM 2 + SCOP) as well as in ovariectomized rats (OVX + SCOP) as compared to the age-matched normal rats (SHAM 1). Apart from the 50 mg/kg dose of *F. umbellata* aqueous extract, all treatments induced a significant increase in the percentage of spontaneous alternations as compared to ovariectomized rats (OVX + SCOP).

### 3.4. Effect of 7-Methoxycoumarin and *F. umbellata* Extract on Long-Term Memory

**Figure 5:**
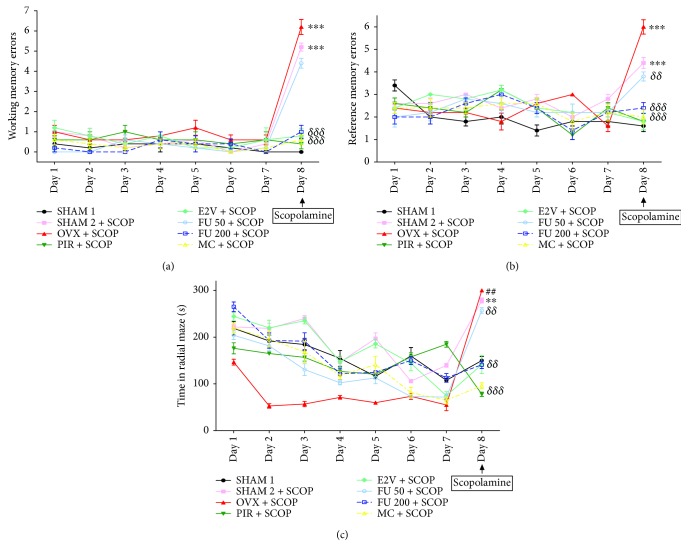
Graphic representation of the working memory (a) and spatial reference memory (b) errors as well as the time past in radial maze (c) before and after scopolamine exposition in rats treated with different substances into the Y-maze. SHAM 1 = sham-operated rats treated with vehicle as normal control 1; SHAM 2 = sham-operated rats treated with vehicle as normal control 2; OVX = ovariectomized rats treated with vehicle as negative control; PIR = ovariectomized rats treated with piracetam at the dose of 1.5 mg/kg BW as positive control 1; E2V = ovariectomized rats treated with estradiol valerate at the dose of dose 1 mg/kg BW as positive control 2; FU 50 and 200 = ovariectomized rats treated with *F. umbellata* aqueous extract at the doses of 50 and 200 mg/kg BW, respectively; MC = ovariectomized rats treated with 7-methoxycoumarin at the dose of 1 mg/kg BW. SCOP = animal that received a single dose of scopolamine (2 mg/kg BW). ^∗∗^ *p* < 0.01 and ^∗∗∗^ *p* < 0.001 as compared to the SHAM 1 group; ^##^ *p* < 0.01 as compared to the SHAM 2 group; ^*δδ*^ *p* < 0.01 and ^*δδδ*^ *p* < 0.001 as compared to the OVX group.

The radial arm maze test was performed for 8 days, from the 14th to the 21st days of treatment. It was noted that before the scopolamine-induced memory impairment, the ovariectomized rats (OVX) showed more working (Figure 5(a)) and spatial reference memory (Figure 5(b)) errors as compared to normal rats (SHAM 2), but the difference was not statistically significant. After scopolamine administration, normal (SHAM 2) and ovariectomized rats displayed more (*p* < 0.001) working and spatial reference memory errors as compared to normal rats, which did not receive scopolamine (SHAM 1). The ovariectomized rats treated with *F. umbellata* aqueous extract (50 and 200 mg/kg) as well as MC (1 mg/kg) showed a significant (*p* < 0.001) reduction of working memory and spatial reference memory errors as compared to the OVX group.

The variations of the scanning time in the radial maze with different groups are depicted in Figure 5(c). It can be observed that ovariectomized rats (OVX) are faster in the radial arm maze before scopolamine injection as compared to all other groups. Moreover, the time required to collect all the baits in the radial arm maze gradually decreased from day 1 to day 7 in rats treated with the *F. umbellata* aqueous extract at both doses as well as with MC. Scopolamine administration on day 8 significantly (*p* < 0.01) increased the time taken to collect all baits by ovariectomized (OVX + SCOP) and normal (SHAM 2 + SCOP) rats as compared to the normal control group (SHAM 1). However, ovariectomized rats treated with *F. umbellata* aqueous extract (200 mg/kg) and MC (1 mg/kg) showed a significant (*p* < 0.01) reduction in this parameter as compared to OVX rats.

### 3.5. Effect of 7-Methoxycoumarin and *F. umbellata* Extract on Anxiety Parameters

**Figure 6:**
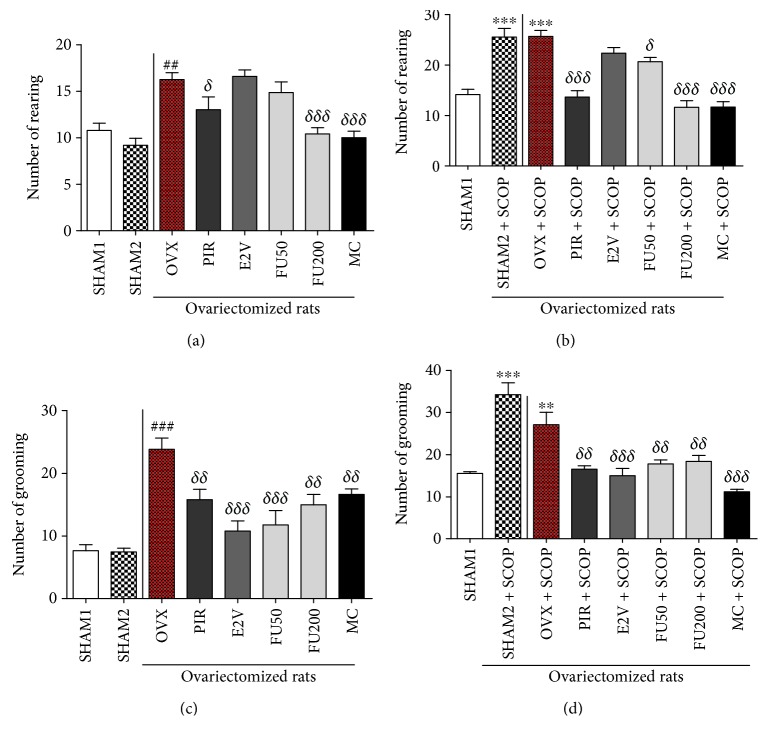
Graphic representation of the number of rearing (a and b) and grooming (c and d) of rats in the Y-maze before and after scopolamine exposition in rats treated with different substances into the Y-maze. SHAM 1 = sham-operated rats treated with vehicle as normal control 1; SHAM 2 = sham-operated rats treated with vehicle as normal control 2; OVX = ovariectomized rats treated with vehicle as negative control; PIR = ovariectomized rats treated with piracetam at the dose of 1.5 mg/kg BW as positive control 1; E2V = ovariectomized rats treated with estradiol valerate at the dose of 1 mg/kg BW as positive control 2; FU 50 and 200 = ovariectomized rats treated with *F. umbellata* aqueous extract at the doses of 50 and 200 mg/kg BW, respectively; MC = ovariectomized rats treated with 7-methoxycoumarin at the dose of 1 mg/kg BW. SCOP = animal that received a single dose of scopolamine (2 mg/kg BW). ^∗∗^ *p* < 0.01 and ^∗∗∗^ *p* < 0.001 as compared to the SHAM 1 group; ^##^ *p* < 0.01 and ^###^ *p* < 0.001 as compared to the SHAM 2 group; ^*δ*^ *p* < 0.05, ^*δδ*^ *p* < 0.01, and ^*δδδ*^ *p* < 0.001 as compared to the OVX group.

It can be observed in Figures 6(a) and 6(c) that ovariectomy induced a significant increase in the number of rearing (*p* < 0.01) and grooming (*p* < 0.001) as compared to normal rats. There was a significant decrease in the number of rearing with piracetam (*p* < 0.05), *F. umbellata* at the dose of 200 mg/kg (*p* < 0.001), and MC (*p* < 0.001), whereas the number of grooming was significantly reduced with piracetam (*p* < 0.01), estradiol (*p* < 0.001), *F. umbellata* 50 mg/kg (*p* < 0.001) and 200 mg/kg (*p* < 0.01), and MC (*p* < 0.01) treatment. Following the administration of scopolamine, there was a significant (*p* < 0.001) increase in the number of rearing as well as grooming in normal rats (SHAM 2 + SCOP) and ovariectomized (OVX + SCOP) rats as compared to normal rats (Figures 6(b) and 6(d)) which did not receive scopolamine (SHAM 1). Piracetam, *F. umbellata* aqueous extract at all tested doses, and MC significantly decreased the number of rearing and grooming. E2V (1 mg/kg) induced a significant reduction only in the number of grooming as compared to the OVX group.

### 3.6. Effects on the Weight and the Microarchitecture of the Brain

**Figure 7:**
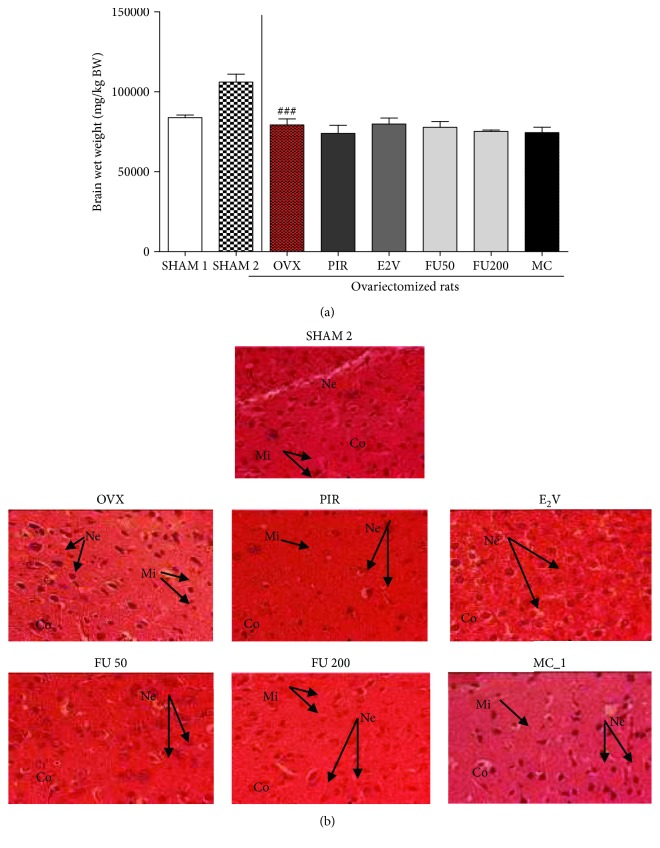
Graphical representation of brain wet weight (a) and microphotographs of HE-stained sections (400x) of the hippocampal region of the brain (b) from different experimental rat groups in postmenopause-like conditions after 3 weeks of treatment. SHAM 1 = sham-operated rats treated with vehicle as normal control 1; SHAM 2 = sham-operated rats treated with vehicle as normal control 2; OVX = ovariectomized rats treated with vehicle as negative control; PIR = ovariectomized rats treated with piracetam at the dose of 1.5 mg/kg BW as positive control 1; E2V = ovariectomized rats treated with estradiol valerate at the dose of dose 1 mg/kg BW as positive control 2; FU 50 and 200 = ovariectomized rats treated with *F. umbellata* aqueous extract at the doses of 50 and 200 mg/kg BW, respectively; MC = ovariectomized rats treated with 7-methoxycoumarin at the dose of 1 mg/kg BW. Mi = microglia; Ne = neurons; Co = cortex. ^###^ *p* < 0.001 as compared to SHAM 2 group.

A significant (*p* < 0.01) decrease in the brain wet weight was observed in ovariectomized rats as compared to normal rats (Figure 7(a)). However, no significant changes were noted following treatment with the different tested substances in this parameter. No alterations in the microarchitecture of hippocampal brain regions were noted in this work (Figure 7(b)).

### 3.7. Effects on Oxidative Stress Parameters

**Figure 8:**
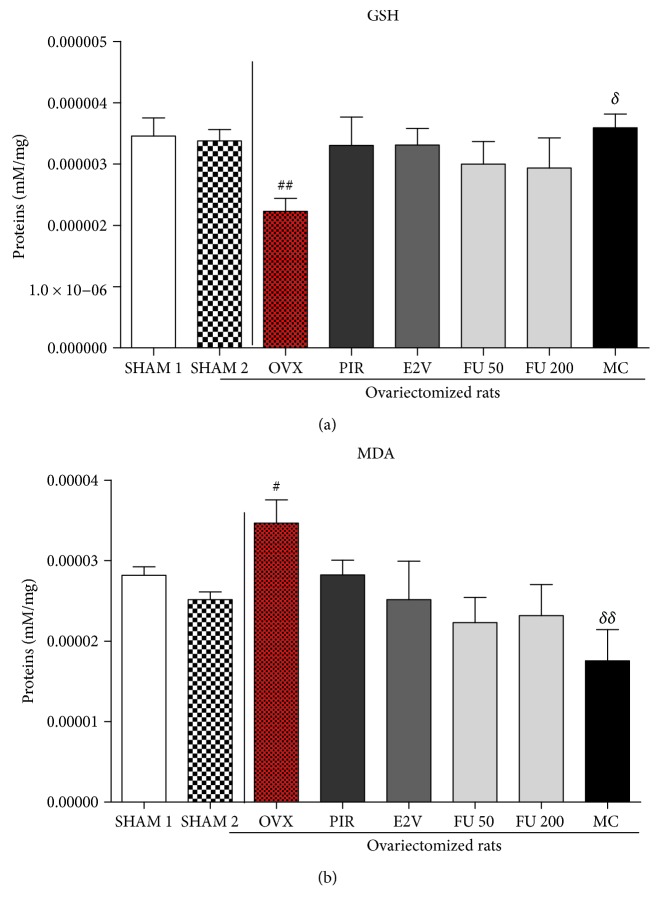
Reduction in glutathione-GSH (a) and malondialdehyde-MDA (b) after scopolamine exposition in rats treated with different substances. SHAM 1 = sham-operated rats treated with vehicle as normal control 1; SHAM 2 = sham-operated rats treated with vehicle as normal control 2; OVX = ovariectomized rats treated with vehicle as negative control; PIR = ovariectomized rats treated with piracetam at the dose of 1.5 mg/kg BW as positive control 1; E2V = ovariectomized rats treated with estradiol valerate at the dose of dose 1 mg/kg BW as positive control 2; FU 50 and 200 = ovariectomized rats treated with *F. umbellata* aqueous extract at the doses of 50 and 200 mg/kg BW, respectively; MC = ovariectomized rats treated with 7-methoxycoumarin at the dose of 1 mg/kg BW. ^#^ *p* < 0.05 as compared to SHAM 1; ^##^ *p* < 0.01 as compared to the SHAM 2 group; ^*δ*^ *p* < 0.05 and ^*δδ*^ *p* < 0.01 as compared to the OVX group.

Figure 8 depicts the effects of treatments on MDA and GSH levels in the brain's homogenate. A decrease in GSH level (*p* < 0.01) and an increase in MDA level (*p* < 0.05) were observed in the OVX group as compared to the sham-operated group following scopolamine administration. All treated groups prevented a decrease in GSH and an increase in MDA, but only the MC group reached statistical significant level (*p* < 0.05 for GSH and *p* < 0.01 for MDA).

## 4. Discussion

When women achieve menopause, they are challenged by the effects of estrogen loss on energy, mood, cognitive function, and memory. These stresses are compounded by increased risks for cardiovascular diseases, osteoporosis, cancer, and neurodegenerative diseases [1]. One of the most common diseases among the elderly population is AD, which is classified as a highly complex and progressive neurodegenerative disease [5]. AD is the main cause of dementia worldwide and is characterized by memory decrease and cognitive dysfunction [30]. Two main hypotheses explain the morphological and biochemical changes in AD patients: amyloid and cholinergic hypothesis [31]. The “cholinergic hypothesis” is characterized by a decrease in acetylcholine (ACh) in cholinergic neurons present in the hippocampus and cerebral cortex [32]. The scopolamine-induced memory impairment rat model, which mimics certain aspects of AD-related cognitive impairment and dementia, is based on the cholinergic hypothesis. In fact, scopolamine is a nonselective antagonist of the muscarinic receptor which is capable of blocking cholinergic transmission causing cognitive and memory dysfunction, leading to learning and memory deficits [26, 33]. Scopolamine-induced AD-like conditions in ovariectomized rats have long been used to demonstrate the neuroprotective effects of xenobiotic substances [34, 35]. However, none of these studies lasted 84 days after ovariectomy, which is known to be the required time for the setup of postmenopause-like conditions in rats [19–21].

In this study, we demonstrated that subchronic treatment with *F. umbellata* aqueous extract and 7-methoxycoumarin significantly improved memory and decreased rearing and grooming in scopolamine-demented ovariectomized rats. The Y-maze and the radial arm maze are well-characterized hippocampus-dependent spatial memory tasks used in this study. The Y-maze task is a specific and sensitive test of spatial recognition memory in rodents, which is suitable for evaluating memory as it highlights the ability of rodents to explore a novel environment using their hippocampi mainly [36, 37]. Short-term memory was then assessed through spontaneous alternation behavior in the Y-maze as previously described [23, 27, 38]. In the first set of tests, the percentage of spontaneous alternations was found to increase with age. Moreover, a 3-month endogenous estrogen depletion following ovariectomy significantly decreased the percentage of spontaneous alternations. These results suggest that rats gaining insurance with age are more stable in the maze. On the other hand, anxiety parameters significantly decreased with age, but increased with ovariectomy in the Y-maze. The aforementioned results suggest that ovariectomy negatively impacts locomotor activity in rats, making them display anxious behavior. Curiously, ovariectomized rats were quite rapid in the collection of baits in the radial maze, but this performance was accompanied with a high number of working memory errors which decreased with habituation. This can be explained by the great appetite that sets up with ovariectomy. These results are consistent with those of Fader et al. [34], who reported that ovariectomy increased the number of working memory errors in the radial arm maze. Studies on both humans and animals indicate that estrogens may also improve memory in women with Alzheimer's disease and reduce the incidence rate of the disease [39]. Moreover, thirty minutes after scopolamine injection, the number of entries increased while the percentage of spontaneous alternations decreased in rats as compared to normal rats which did not receive scopolamine. *F. umbellata* aqueous extract on the other hand is effective on memory deficits induced by ovariectomy; it loses its promnesic efficacy when ovariectomy is coupled to muscarinic neurotransmission failure. This suggests that the *F. umbellata* extract active components could act upon muscarinic receptors to enhance memory. The failure to do so was due to the presence of scopolamine acting as an antagonist.

The 8-arm radial maze task is widely used in evaluating the effect of drugs, stress, and various other environmental factors on learning and memory [40]. Visual cues set up at the same position throughout the radial maze task acted as spatial references as they are associated with long-term memory and allow the animals to remember the location of the baited arms in the maze. The short-term memory associated with the hippocampus can keep records only for a few seconds to minutes. Memory contents can be transferred, particularly through exercises in the long-term memory [41, 42]. In the second set of tests, working memory errors significantly reduced with age, whereas ovariectomy increased this parameter. It appears from these results that ovariectomy negatively affects the short-term memory by causing anterograde amnesia [41]. Treatment with *F. umbellata* aqueous extract at the dose of 200 mg/kg as well as MC at the dose of 1 mg/kg protected rats against scopolamine-induced memory impairment as evidenced by significantly increased spontaneous alternations, reflecting an improvement in short-term memory. These results suggest that *F umbellata* and its major compound are endowed with neuroprotective activities given that working memory and reference memory are variables that report the physiological status of the brain [42]. It has been reported that estrogen administered to ovariectomized rats improves working memory performance during acquisition of the radial arm maze task with four arms baited and prevents the disruptive effects of scopolamine on that task [34]; these findings are consistent with ours. The prevention by estrogen of impairments in working memory performance following scopolamine administration has been attributed to the ability of estrogen to bolster cholinergic functioning in the hippocampus by increasing the activity of choline acetyltransferase [43]. Authors also hypothesized that estrogen may act as growth factors for cholinergic neurons [44] to facilitate reactive fiber growth in hippocampi of ovariectomized rats [45]. Since *F. umbellata* contains phytoestrogens, it can act like estrogen in hippocampi and then counteract the effects of scopolamine. *F. umbellata* aqueous extract components could protect rats against memory impairment by binding on estrogen receptors (ER*α* and ER*β*) found in several brain regions including the hippocampus, thereby inhibiting cholinesterase increase induced by scopolamine [46]. The UHPLC-MS performed with this extract revealed that *F. umbellata* contains genistein and biochanin A, well-characterized phytoestrogens [18]. Indeed, previous scientific reports showed that soy isoflavones (daidzein and genistein) improved learning ability and memory of rodents [47, 48] as well as humans [49, 50]. These activities were attributed to the ability of soy isoflavones to mimic the effects of endogenous estrogens through estrogen receptor *β* in the brain [51]. So far, 7-methoxycoumarin failed to transactivate ER*β* such as *F. umbellata* extracts in HEK293T cells stably transfected with ER*β* in a reporter gene assay [17]; it induced its estrogenicity via a nongenomic pathway [18]. The observed neuroprotective effects of *F. umbellata* might be due to the presence of genistein or related chemicals in this plant. Although MC failed to transactivate ER*β*; coumarins are well known as selective inhibitors of acetylcholinesterase [52, 53]. MC could therefore pass through a nongenomic pathway in the brain to increase acetylcholine concentration, thereby increasing nerve impulses [54]. Anxiety is a parameter that negatively affects learning and memory. The injection of scopolamine helps to accentuate the disorder in rats. The treatments with *F. umbellata* aqueous extract at doses of 50 and 200 mg/kg as well as MC decreased the number of rearing as well as grooming in rats. A chemical messenger widespread in the brain is GABA, which reduces activity of neurons. Some researchers believe that GABA would serve among other things to control the fear or anxiety manifested by neuronal excitation [55]. Scopolamine-induced memory deficits are accompanied with changes in acetylcholinesterase activity [56]. *F. umbellata* aqueous extract and MC exhibited anxiolytic properties and act as GABA agonists that bind to receptor GABAergic neurons and modulating the activity of the nervous system [55].

It is well known that scopolamine-induced memory deficits are accompanied with oxidative damage [57]. A decrease in GSH level and an increase in MDA level in OVX rats were observed after scopolamine administration as compared to sham-operated rats. GSH is an important intracellular radical scavenger, a substrate of many xenobiotic elimination reactions, and participates in the elimination of reactive intermediates by reducing hydroperoxides [58, 59]. The depletion of GSH levels leads to the increase in oxidative stress. MC prevented the scopolamine-induced accumulation of hydroperoxides by increasing GSH levels. In addition, MDA levels are a lipid peroxidation biomarker. The main target of reactive oxygen species (ROS) is the polyunsaturated fatty acid in cell membranes. They cause lipid peroxidation and formation of MDA, which may lead to damage of cell structure and function [60]. MC reduced MDA levels as compared to the OVX group in this study. These results suggest a reduction of LDL peroxidation. From this result, the neuroprotective effects of MC may be attributable to its possible antioxidant activity.

## 5. Conclusion

As *F. umbellata* aqueous extract, 7-methoxycoumarin significantly increased spontaneous alternation and decreased the working and spatial reference memory errors, the time taken to collect the baits in the 8-arm radial maze, and the rearing and grooming in ovariectomized rats. Taken altogether, the aforementioned results suggest that 7-methoxycoumarin is endowed with potential neuroprotective effects in rats. In addition, MC showed an antioxidant activity, which could account for its neuroprotective effects. 7-Methoxycoumarin is responsible at least in part for the neuroprotective effects of *F. umbellata*.

## Abbreviations

C-hex: Cyclohexane
CPC: Centrifugal partition chromatography
DCM: Dichloromethane
DMSO: Dimethylsulfoxide
ER: Estrogen receptor
F: Fraction
CNH: Cameroon National Herbarium
HPLC: High-performance liquid chromatography
MC: 7-Methoxycoumarin
MeOH: Methanol
NMR: nuclear magnetic resonance.

## Supplementary Materials

Supplementary Materials Supplementary Figures AD.
