# Supplementary material for: Effects of Ficus umbellata (Moraceae) Aqueous Extract and 7-Methoxycoumarin on Scopolamine-Induced Spatial Memory Impairment in Ovariectomized Wistar Rats
Source: Behav Neurol. 2018 Sep 30;2018:5751864. doi: 10.1155/2018/5751864 (PMC6186347; doi:10.1155/2018/5751864)
Supplement: Supplementary Materials — Supplementary Figures AD. [file 5751864.f1.docx]

**7-methoxycoumarin**

Supplementary data 1

Supplementary data 2

| Tr | m/z | EC | ppm | DBE | Fragments (m/z, EC, DBE) |
| --- | --- | --- | --- | --- | --- |
| 2.16 | 463.1452  927.2968 [2M-H]^-^ | C_19_H_27_O_13_  C_38_H_55_O_26_ | -0.6 | 8.5 |  |
| 2.32 | 233.1022 | C_10_H_17_O_6_ | -1.3 | 2.5 |  |
| 2.41 | 241.0707 | C_11_H_13_O_6_ | -2.1 | 5.5 |  |
| 2.52 | 447.1500  493.1559 [M-H+HCOOH]^-^ | C_19_H_27_O_12_  C_20_H_29_O_14_ | -0.7  0.4 | 6.5  6.5 |  |
| 2.91 | 477.1604  523.1663 [M-H+HCOOH]^-^ | C_20_H_29_O_13_  C_21_H_31_O_15_ | -0.8  0 | 6.5  6.5 |  |
| 3.07 | 461.1653  507.1708 [M-H+HCOOH]^-^ | C_20_H_29_O_12_  C_21_H_31_O_14_ | 1.3  -1.2 | 6.5  6.5 | 329.1219, C_15_H_21_O_8_, 5.5  271.0808, C_12_H_15_O_7_, 5.5  161.0444, C_6_H_9_O_5_, 2.5 |
| 3.50 | 517.2291  563.2346 [M-H+HCOOH]^-^ | C_24_H_37_O_12_  C_25_H_39_O_14_ | 1.1  1.2 | 6.5  6.5 | 387.2017, C_19_H_31_O_8_, 4.5 |
| 3.34 | 491.1759  537.1820 [M-H+HCOOH]^-^ | C_21_H_31_O_13_  C_22_H_33_O_15_ | -1.2  0.2 | 6.5  6.5 | 359.1330, C_16_H_23_O_9_, 5.5 |
| 3.46 | 549.2914  595.2975 [M-H+HCOOH]^-^ | C_26_H_45_O_12_  C_27_H_47_O_14_ | 1.2  1.5 | 4.5  4.5 | 417.2460, C_21_H_37_O_8_, 3.5 |
| 3.55 | 211.0607 | C_10_H_11_O_5_ | 0.5 | 5.5 | 193.0496, C_10_H_9_O_4_, 6.5  165.0552, C_9_H_9_O_3_, 5.5  149.0600, C_9_H_9_O_2_, 5.5 |
| 3.75 | 713.1927 | C_31_H_37_O_19_ | -0.3 | 13.5 | 567.1350, C_25_H_27_O_15_, 12.5  545.2609, C_22_H_41_O_15_, 2.5  399.0917, C_17_H_19_O_11_, 8.5 |
| 4.34 | 263.0919 | C_14_H_15_O_5_ | 0 | 7.5 | 175.0394, C_10_H_7_O_3_, 7.5  147.0447, C_9_H_7_O_2_, 6.5 |
| 4.57 | 265.1074 | C_14_H_17_O_5_ | -0.2 | 6.5 | 221.1174, C_13_H_17_O_3_, 5.5  163.0753, C_10_H_11_O_2_, 4.5 |
| 4.73 | 269.1024 | C_13_H_17_O_6_ | -0.4 | 5.5 | 195.0650, C_10_H_11_O_4_, 5.5  151.0753, C_9_H_11_O_2_, 4.5 |
| 4.88 | 387.1076  775.2242 [2M-H]^-^ | C_20_H1_9_O_8_  C_40_H_39_O_16_ | -0.4  -3.1 | 11.5  12.5 | 369.0969, C_20_H_17_O_7_, 12.5  325.1072, C_19_H_15_O_5_, 11.5 |
| 5.75 | 531.2808  577.2866 [M-H+HCOOH]^-^ | C_26_H_43_O_11_ C_27_H_45_O_13_ | 0.8  1 | 5.5  5.5 | 399.2375, C_21_H_35_O_7_, 4.5  167.0341, C_8_H_7_O_4,_ 5.5 |
| 6.66 | 369.0970  739.2011 [2M-H]^-^ | C_20_H_17_O_7_  C_40_H_35_O_14_ | -1.1 | 12.5 | 325.1076, C_19_H_17_O_5_, 11.5  271.0964, C_16_H_15_O_4_, 9.5  189.0549, C_11_H_9_O_3_, 7.5  149.0602, C_9_H_9_O_2_, 5.5 |
| 6.78 | 401.1238 | C_21_H_21_O_8_ | 0.5 | 11.5 | 369.0971, C_20_H_17_O_7_, 12.5  189.0549, C_11_H_9_O_3_, 7.5  149.0602, C_9_H_9_O_2_, 5.5 |
| 8.28 | 383.1130  207.0654 (maj) | C_21_H_19_O_7_  C_11_H_11_O_4_ | -0.3  -1.4 | 12.5  6.5 | 175.0387, C_10_H_7_O_3_, 7.5  161.0238, C_9_H_5_O_3_, 7.5  147.0439, C_9_H_7_O_2_, 6.5 |
| 8.50 | 611.3797 | C_33_H_55_O_10_ | 0 | 6.5 | 453.2492, C_24_H_37_O_8_, 6.5  391.2491, C_23_H_35_O_5_, 6.5  329.2332, C_18_H_33_O_5_, 2.5  303.1965, C_19_H_27_O_3_, 6.5 |
| 17.74 | 281.2848  327.2900 [M-H+HCOOH]^-^ | C_19_H_37_O C_20_H_39_O_3_ | 0.3  0.2 | 1.5  1.5 |  |
| 18.29 | 295.2996  341.3052 [M-H+HCOOH]^-^ | C_20_H_39_O  C_21_H_41_O_3_ | 0.2  -0.9 | 1.5  1.5 |  |
